# Seasonal particulate matter exposure is associated with upper respiratory microbiota restructuring in dairy heifers

**DOI:** 10.1186/s12917-026-05580-y

**Published:** 2026-05-22

**Authors:** Songphon Buddhasiri, Thaya Muangplod, Peetiporn Panathiwat, Pornpan Chandrasrimuang, Jakree Jitjumnong, Sukolrat Boonyayatra, Tawatchai Singhla

**Affiliations:** 1 Veterinary Public Health and Food Safety Centre for Asia Pacific, Faculty of Veterinary Medicine, Chiang Mai University, Chiang Mai, Thailand; 2 Faculty of Veterinary Medicine, Chiang Mai University, Chiang Mai, Thailand; 3 Department of Animal and Aquatic Sciences, Faculty of Agriculture, Chiang Mai University, Chiang Mai, Thailand; 4 Department of Veterinary Clinical Sciences, Lewyt College of Veterinary Medicine, Long Island University, Brookville, NY USA; 5 Research Center for Veterinary Biosciences and Veterinary Public Health, Faculty of Veterinary Medicine, Chiang Mai University, Chiang Mai, Thailand

**Keywords:** Respiratory Microbiome, Bovine Respiratory Disease, Particulate Matter, Dairy Cattle, Air Pollution

## Abstract

**Background:**

Particulate matter (PM), particularly PM2.5 and PM10, is a major environmental health concern linked to respiratory diseases in humans and animals. Northern Thailand, especially Chiang Mai, experiences recurrent seasonal air pollution from biomass burning, exposing outdoor-housed livestock to elevated ambient PM levels. The bovine upper respiratory tract (URT) harbors both commensal and opportunistic microorganisms, and disruption of this microbiota may influence susceptibility to bovine respiratory disease (BRD). However, the impact of natural PM exposure on the bovine URT microbiota remains poorly understood.

**Results:**

Nasopharyngeal swabs from 25 clinically healthy dairy heifers were analyzed during low-PM and high-PM periods. During the high-PM period, peak PM2.5 levels exceeded 30 times the WHO 24-hour guideline. Alpha diversity, including observed features, Shannon diversity, and Simpson index, was significantly higher during the high-PM period than during the low-PM period. Beta diversity analysis showed significant differences in Bray–Curtis dissimilarity and Jaccard distance, indicating changes in both relative abundance-based community structure and presence–absence-based community membership. The high-PM period was characterized by altered taxonomic profiles, including higher proportions of Proteobacteria, Firmicutes, Gammaproteobacteria, and Bacilli, and lower proportions of Actinobacteriota, Bacteroidota, Actinobacteria, and Bacteroidia. Among selected dominant genera, *Moraxella* and *Fusobacterium* were significantly reduced during the high-PM period. BRD-associated genera, including *Mycoplasma*, *Pasteurella*, *Mannheimia*, and *Histophilus*, showed higher average relative abundances during the high-PM period; however, paired comparisons were not statistically significant.

**Conclusions:**

Seasonal high-PM exposure in Chiang Mai was associated with measurable changes in the nasopharyngeal microbiota of clinically healthy dairy heifers, including increased alpha diversity, altered beta diversity, changes in taxonomic profiles, and reductions in selected dominant genera. These findings suggest that ambient air pollution may contribute to respiratory microbiota restructuring in dairy heifers. Further longitudinal studies integrating microbiota composition, host immune responses, farm-level environmental monitoring, and clinical respiratory outcomes are needed to clarify whether PM-associated microbiota changes contribute to BRD susceptibility.

**Supplementary Information:**

The online version contains supplementary material available at 10.1186/s12917-026-05580-y.

## Background

Particulate matter (PM), including fine particles (PM2.5) and coarse particles (PM10), is a significant environmental health concern worldwide [1]. These airborne particles originate from both natural sources and anthropogenic activities, such as industrial emissions, vehicular exhaust, and biomass burning [2–4]. Both chronic and acute exposure to PM has been linked to a wide range of adverse health outcomes, including respiratory and cardiovascular diseases, oxidative stress, and inflammation in humans and animals [5–9].

Northern Thailand, particularly Chiang Mai province, has experienced recurrent air pollution crises for over a decade, with annual peaks in PM concentrations during the dry season from January to April [10–14]. Seasonal biomass burning, primarily from agricultural residues and forest fires, is a major contributor to this decline in air quality [15, 16]. This environmental challenge affects not only human populations but also livestock raised in open-air systems, which are directly exposed to airborne pollutants.

The respiratory microbiota plays a crucial role in maintaining mucosal homeostasis and host defense [17, 18]. In cattle, the upper respiratory tract (URT), particularly the nasopharynx, serves as a reservoir for both commensal and opportunistic microorganisms [19]. Disruption of this microbial community may predispose animals to bovine respiratory disease (BRD), a multifactorial syndrome driven by environmental stress, immune dysregulation, viral pathogens, and the overgrowth of opportunistic pathogens, including *Mycoplasma bovis*, *Pasteurella multocida* (*P. multocida*), *Mannheimia haemolytica* (*M. haemolytica*), and *Histophilus somni* [20–22]. Although these bacteria may be present in the URT of clinically healthy cattle, environmental stressors can disrupt respiratory microbial balance and may favor conditions that allow opportunistic bacteria to become more abundant or pathogenic [20, 22, 23].

Previous studies in humans and experimental animal models suggest that PM exposure can alter respiratory microbial diversity and community composition, disrupt host–microbe interactions, and increase susceptibility to respiratory inflammation or infection [24–27]. For example, PM exposure has been associated with shifts in airway or pulmonary microbiota structure in humans, rodents, and poultry, indicating that airborne particles may act as environmental stressors capable of reshaping respiratory microbial communities. However, few studies have investigated the impact of PM on the bovine respiratory microbiota under natural field conditions. To date, no study has assessed the effect of ambient PM exposure on the URT microbiota of dairy cattle in Thailand, a region that experiences recurrent seasonal air pollution. Therefore, this study aimed to compare the nasopharyngeal microbiota of clinically healthy dairy heifers between low-PM and high-PM periods during the dry season in Chiang Mai. Specifically, we aimed to assess changes in microbial alpha and beta diversity, characterize shifts in taxonomic composition, and evaluate selected genera associated with BRD.

## Methods

### Study design

**Fig. 1:**
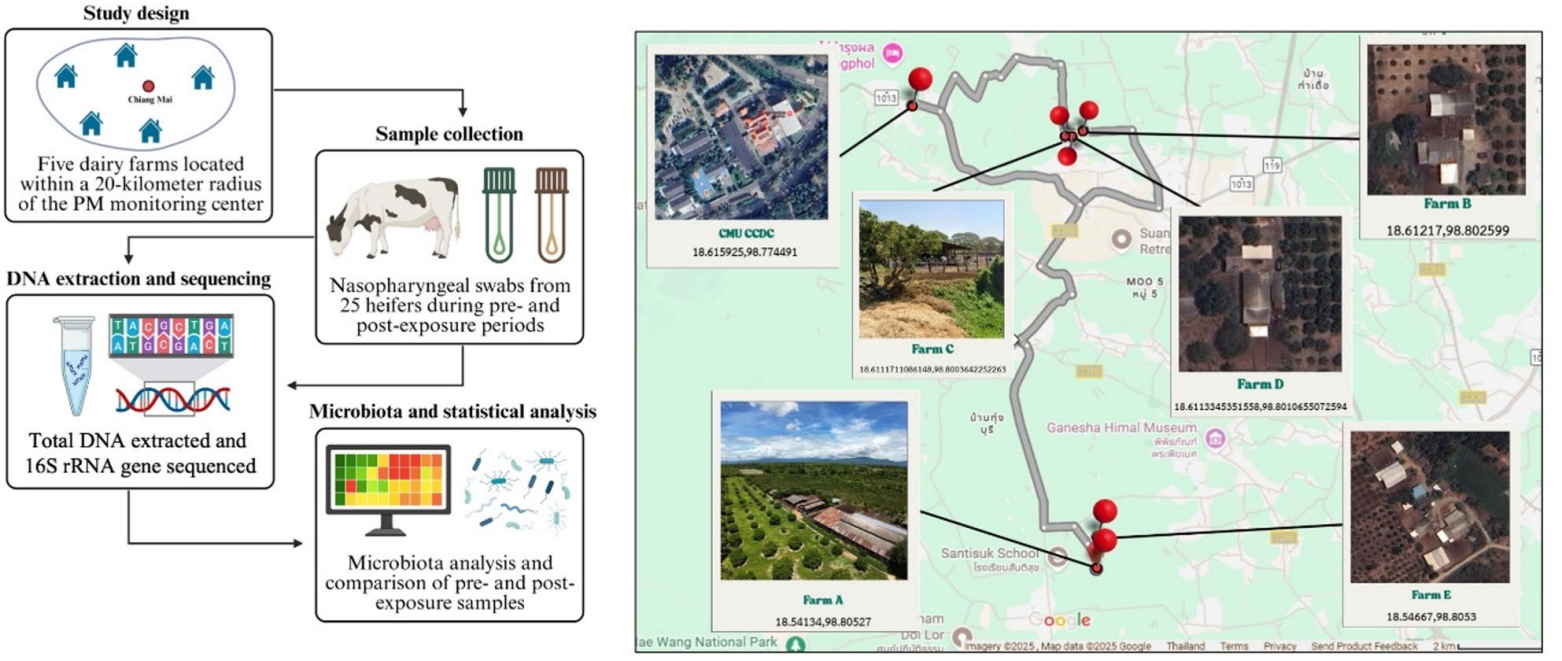
Schematic overview of the study design for assessing the association between seasonal PM exposure and the nasopharyngeal microbiota of dairy heifers, including sampling farm locations and the PM monitoring center (CMU CCDC)

A longitudinal cohort study with a repeated-measures design was conducted on dairy farms in Chiang Mai, Thailand. Five farms located within a 10-kilometer radius of the PM monitoring center were selected (Fig. 1). To minimize potential confounding effects, farms with broadly similar management systems were included. Heifers were housed in open-sided barns with natural ventilation, which is a common housing system in the region. Animals were fed roughage-based diets, including forage and/or corn silage, supplemented with concentrate feed. Routine herd health management, including deworming and vaccination programs, was implemented according to local veterinary recommendations. Bedding materials and general hygiene practices were broadly similar among farms. However, detailed quantitative measurements of barn-level airflow, bedding-derived particulates, feed dust, and indoor particulate concentrations were not performed.

Replacement dairy heifers were selected because this group represents a relevant population for investigating early respiratory microbiota responses during the seasonal PM period and because their inclusion reduced potential confounding associated with adult lactating cows, including parity, lactation status, pregnancy, metabolic stress, and longer cumulative production history. Clinically healthy dairy heifers with no recorded history of BRD or prior treatment for BRD were enrolled based on farm records and farmer/veterinarian information. All animals underwent a general clinical examination by a veterinarian at the time of sampling. However, subclinical respiratory disease could not be excluded, as diagnostic imaging, such as lung ultrasonography, was not performed.

Heifers were randomly selected from each farm, with 3–6 animals enrolled per farm to include representation across farms within the study area. The same animals were sampled during both the low-PM and high-PM periods, allowing each animal to serve as its own control. A formal a priori sample size calculation was not performed because preliminary effect-size estimates for PM-associated changes in the bovine nasopharyngeal microbiota were not available. The sample size was determined based on feasibility, logistical constraints, and the availability of eligible heifers.

The two-month interval was selected to capture microbiota changes after sustained real-world exposure during the seasonal PM peak. Although no standardized exposure duration has been established for bovine nasopharyngeal microbiota responses to PM, previous studies in dairy cattle and calves have shown that ambient air pollution or wildfire-derived PM2.5 exposure can be associated with measurable immune, physiological, and production-related effects under field conditions [5, 28, 29].

### Sample collection

Nasopharyngeal swabs were collected from 25 heifers using sterile single-guarded swabs (28-inch; J0272, Jorgensen Laboratories, Loveland, CO, USA) at two time points: during the low-PM period in January 2024 and during the high-PM period in April 2024. The low-PM period represented the sampling period before the major seasonal PM peak, whereas the high-PM period represented the sampling period after approximately two months of sustained elevated PM exposure. Swabs were placed into collection tubes containing DNA/RNA Shield (Zymo Research Corporation, CA, USA) to preserve nucleic acids. All samples were transported on ice and delivered to the laboratory within 24 h of collection. Upon arrival, samples were stored at − 80 °C until DNA extraction.

### Data collection

Data on the enrolled heifers, including age and farm location, were recorded. PM2.5 and PM10 concentrations (µg/m³) were obtained from the Climate Change Data Center at Chiang Mai University (CMU CCDC), which monitored air quality at 5-minute intervals using a PM monitoring center [30].

### DNA extraction and 16 S rRNA gene sequencing

Total DNA from nasopharyngeal swab samples was extracted using ZymoBIOMICS DNA Miniprep Kit (Zymo Research Corporation, CA, USA), following the manufacturer’s protocol. Briefly, swabs were cut and placed in bead-beating tubes with lysis solution and homogenized using a bead beater. The lysates were centrifuged and filtered using filter tubes. The filtrates were mixed with the DNA binding buffer and added to spin columns. The columns were washed with buffer and eluted with DNase/RNase free water. DNA was purified using a spin filter. DNA concentration and purity were assessed using a NanoDrop spectrophotometer (Thermo Fisher Scientific, USA). DNA integrity was evaluated by 1% agarose gel electrophoresis. The extracted DNA was stored at − 20 °C until further analysis.

After quality validation, PCR amplification of the V3–V4 region of the 16 S rRNA gene was performed using primer pairs 338 F (ACTCCTACGGGAGGCAGCAG) and 806R (GGACTACHVGGGTWTCTAAT) to classify bacterial taxa [31]. Negative controls were included during both DNA extraction and PCR amplification. For DNA extraction, an extraction negative control was included by placing an unused sterile swab directly into DNA/RNA Shield without contact with an animal sample and processing it alongside the nasopharyngeal swab samples. For PCR amplification, a non-template control (NTC) containing PCR-grade water instead of template DNA was included to monitor PCR reagent contamination. Amplicons were purified for library preparation and subjected to quality control prior to sequencing on the Illumina NovaSeq platform to generate paired-end reads (2 × 250 bp). Following sequencing, raw paired-end reads were subjected to quality-control and bioinformatic processing as described below.

### Microbiota and statistical analysis

Raw paired-end reads were demultiplexed and quality filtered using QIIME 2 (version amplicon 2024.5) [32]. The DADA2 plugin was used to trim low-quality regions, denoise reads, merge paired-end sequences, remove chimeric sequences, and generate amplicon sequence variants (ASVs). A total of 8,725,872 paired-end reads were generated from 50 nasopharyngeal samples, with a median of 138,714.5 input reads per sample and a range of 54,453–860,442 reads per sample. After quality filtering, denoising, paired-end merging, and chimera removal using DADA2, 6,747,327 non-chimeric reads were retained for downstream analysis. The median number of non-chimeric reads per sample was 110,879, with a range of 40,500–746,930 reads per sample. On average, 91.68% of input reads were successfully denoised by DADA2, and 80.28% of input reads were retained as non-chimeric reads. Sample-level DADA2 denoising statistics are provided in Supplementary Table S1. Sequencing depth was assessed using rarefaction analysis (Supplementary Figure S1), and the feature table was rarefied to 40,500 reads per sample for alpha and beta diversity analyses.

Taxonomic classification was performed using a naïve Bayes classifier trained on the SILVA 138 reference database trimmed to the V3–V4 region [33, 34]. ASVs assigned to mitochondria, chloroplasts, archaea, or unclassified at the domain level were excluded from downstream analysis. Relative abundance profiles at multiple taxonomic levels, from phylum to genus, were generated from the ASV table using QIIME 2 and visualized using the microeco R package (version 1.9.1) [35]. Alpha diversity was assessed using observed features, Shannon diversity index, and Simpson index. Beta diversity was calculated using Bray–Curtis dissimilarity, Jaccard distance, unweighted UniFrac, and weighted UniFrac metrics, followed by principal coordinates analysis (PCoA). Differences in beta diversity between the low-PM and high-PM periods were assessed using PERMANOVA. Paired comparisons between low-PM and high-PM samples were performed using the Wilcoxon matched-pairs signed-rank test, with significance defined as P-value < 0.05. No correction for multiple comparisons was applied. Therefore, taxon-level comparisons are interpreted cautiously, and the findings should be validated in larger longitudinal studies.

## Results

### Demographic data, Farm characteristics and PM level

**Fig. 2:**
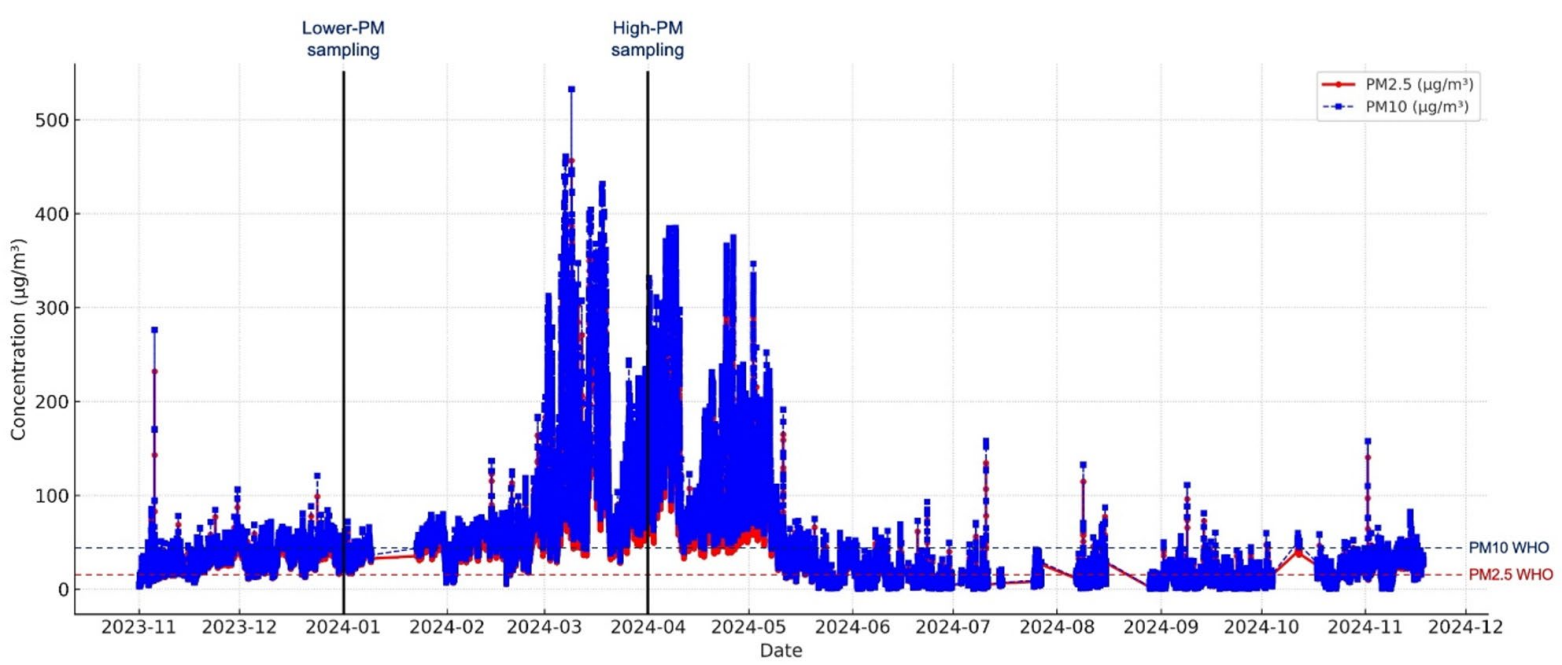
Daily mean concentrations of PM2.5 and PM10 in Mae Wang District, Chiang Mai. Daily average PM2.5 and PM10 concentrations were calculated from continuous air quality monitoring data collected between November 2023 and July 2024. Vertical solid lines indicate the two nasopharyngeal sampling time points during the low-PM and high-PM periods. Horizontal dashed lines represent the WHO 24-hour air quality guideline values for PM2.5 and PM10 at 15 and 45 µg/m³, respectively

From November 2023 onward, ambient particulate matter concentrations, including PM2.5 and PM10, were recorded (Fig. 2). The low-PM sampling was performed in January 2024, when average PM2.5 and PM10 concentrations were 37.69 ± 8.92 µg/m³ and 45.30 ± 12.64 µg/m³, respectively. Between February and April, PM levels peaked sharply, reaching maximum values of 456.47 µg/m³ for PM2.5 and 532.5 µg/m³ for PM10 in March 2024. During this high-PM period, the average PM2.5 and PM10 concentrations were 116.95 ± 68.88 µg/m³ and 133.41 ± 77.24 µg/m³, respectively, coinciding with the dry season and biomass-burning period. The high-PM sampling was performed in April 2024, after approximately two months of elevated PM exposure. PM concentrations subsequently declined after May 2024.

A total of 25 clinically healthy dairy heifers from five farms were enrolled. The number of heifers per farm ranged from 3 to 6, and the farm-level mean age ranged from 10.3 ± 1.83 to 16.7 ± 4.6 months. Overall, the enrolled heifers had a mean age of 13.5 ± 4.2 months. The number of heifers enrolled and the age distribution on each farm are summarized in Table 1.

**Table 1:** Number of enrolled dairy heifers and mean age per farm. Age is presented as mean ± standard deviation (SD)

Farm	Distance from PM sensor (km)	Number of enrolled dairy heifers	Age, months (Mean ± SD)
A	8.95	3	16.4 ± 1.7
B	3.00	5	15.6 ± 4.5
C	2.78	6	16.7 ± 4.6
D	2.85	6	10.3 ± 1.83
E	8.40	5	16.7 ± 4.6
Total		25	13.5 ± 4.2

### Diversity of the respiratory microbiota

**Fig. 3:**
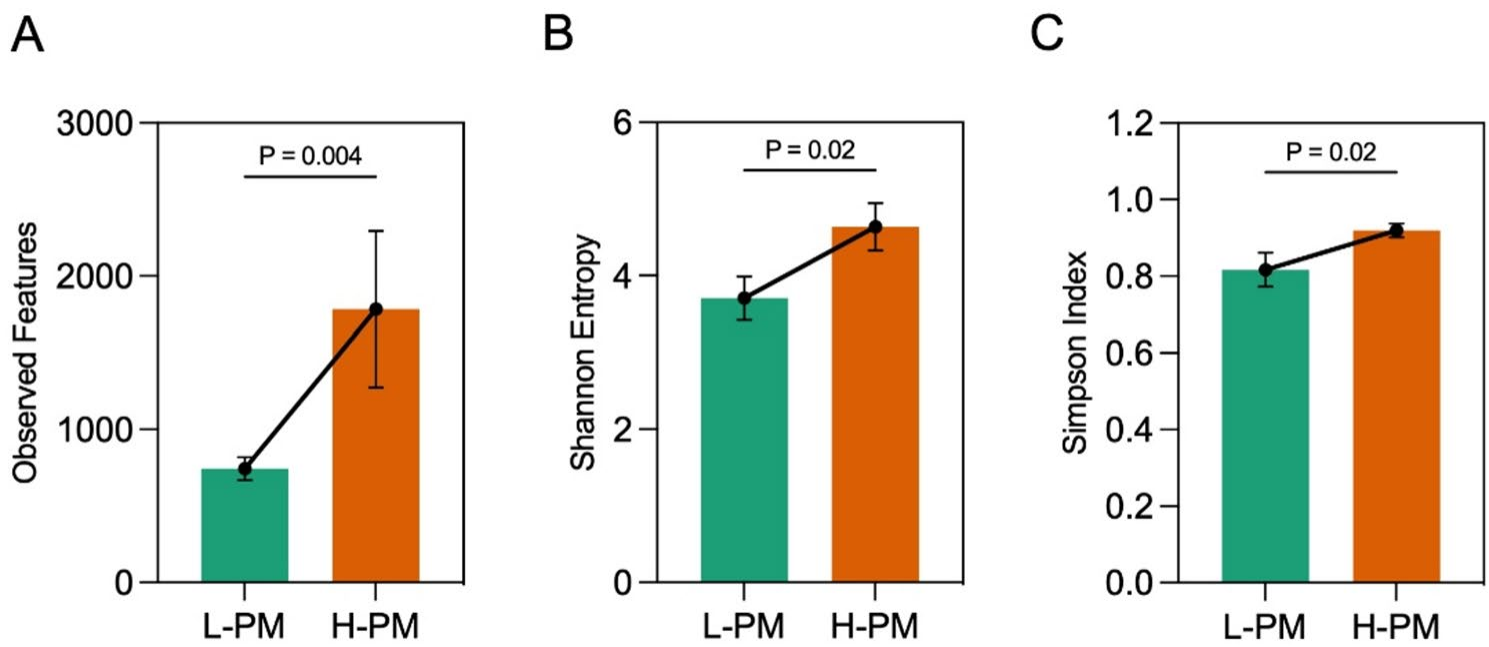
Alpha diversity of the nasopharyngeal microbiota of dairy heifers during the low-PM and high-PM periods. Bar plots show mean ± SEM for (**A**) observed features, (**B**) Shannon diversity index, and (**C**) Simpson index. Paired comparisons between low-PM and high-PM samples were performed using the Wilcoxon matched-pairs signed-rank test, and P values are shown in each panel. L-PM, low-PM period; H-PM, high-PM period

**Fig. 4:**
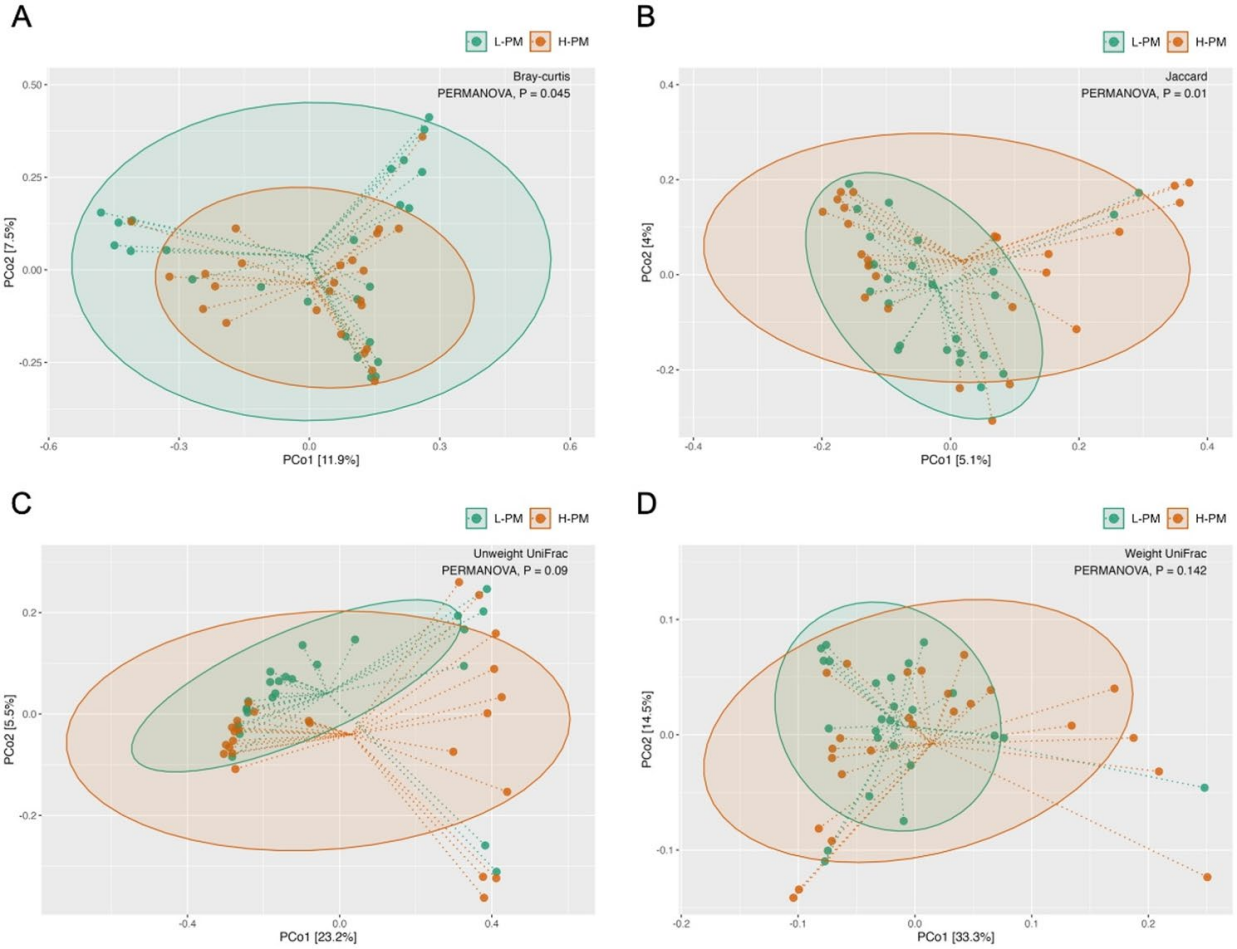
Beta diversity of the nasopharyngeal microbiota of dairy heifers during the low-PM and high-PM periods. Principal coordinates analysis (PCoA) plots were generated based on (**A**) Bray–Curtis dissimilarity, (**B**) Jaccard distance, (**C**) unweighted UniFrac, and (**D**) weighted UniFrac metrics. Differences in community composition between low-PM and high-PM samples were assessed using PERMANOVA. L-PM, low-PM period; H-PM, high-PM period

The respiratory microbiota of dairy heifers during the low-PM and high-PM periods was evaluated using 16 S rRNA gene amplicon sequencing. Alpha diversity was calculated to estimate bacterial diversity within samples, and paired comparisons between low-PM and high-PM samples were performed using the Wilcoxon matched-pairs signed-rank test (Fig. 3). All alpha diversity indices, including observed features, Shannon diversity, and Simpson index, were significantly higher during the high-PM period than during the low-PM period (*P* < 0.05; Figs. 3A–C). Beta diversity was assessed using Bray–Curtis dissimilarity, Jaccard distance, unweighted UniFrac, and weighted UniFrac metrics, followed by PCoA visualization and PERMANOVA testing (Fig. 4). Bray–Curtis dissimilarity and Jaccard distance showed significant differences in community composition between low-PM and high-PM samples (*P* < 0.05; Figs. 4A–B), whereas unweighted UniFrac and weighted UniFrac did not show significant differences.

### Characterization of the respiratory microbiota

**Fig. 5:**
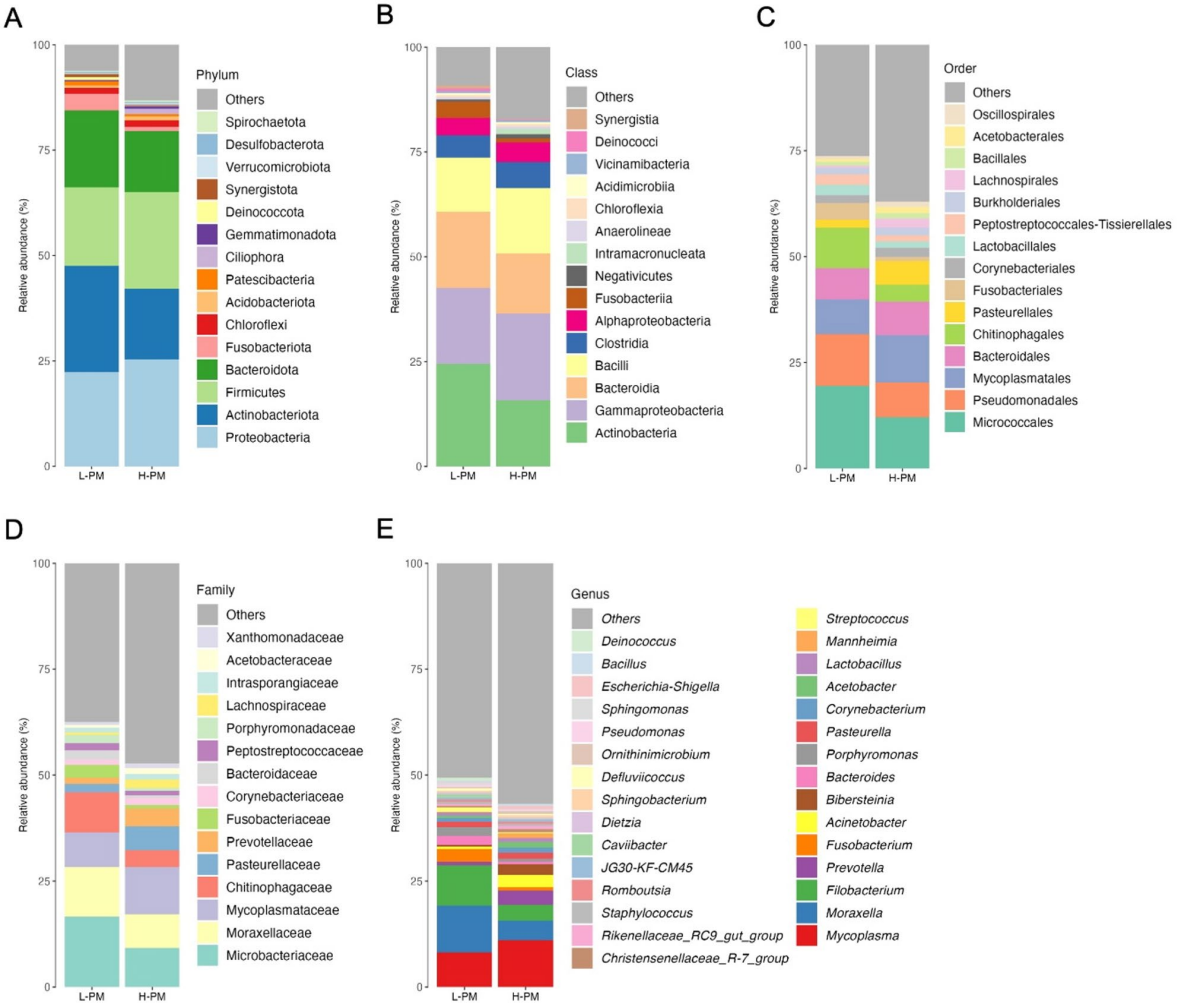
Taxonomic profiles of the nasopharyngeal microbiota of dairy heifers during the low-PM and high-PM periods. Bar plots show the relative abundance of the most abundant bacterial taxa at different taxonomic levels: (**A**) phylum, (**B**) class, (**C**) order, (**D**) family, and (**E**) genus. Each panel compares the average taxonomic composition between low-PM and high-PM samples. L-PM, low-PM period; H-PM, high-PM period

The taxonomic composition of the respiratory microbiota was determined by averaging samples collected during the low-PM and high-PM periods. The number of taxa detected across different taxonomic levels was summarized descriptively to provide an overview of taxonomic breadth during each sampling period (Table 2). At the phylum level, the microbiota in both periods was primarily composed of Actinobacteriota, Proteobacteria, Firmicutes, and Bacteroidota, together accounting for 79–84% of the total microbial community (Fig. 5A). During the low-PM period, the most dominant phyla were Actinobacteriota (25%) and Proteobacteria (22%), followed by Firmicutes (19%) and Bacteroidota (18%). During the high-PM period, Proteobacteria (25%) and Firmicutes (23%) predominated, followed by Actinobacteriota (17%) and Bacteroidota (14%). At the class level, the dominant taxa in both periods included Actinobacteria, Gammaproteobacteria, Bacteroidia, and Bacilli, with smaller contributions from Clostridia, Alphaproteobacteria, and Fusobacteriia (Fig. 5B). During the low-PM period, Actinobacteria (24%) were most abundant, followed by Bacteroidia (18%), Gammaproteobacteria (18%), and Bacilli (13%). During the high-PM period, Gammaproteobacteria (21%) were most abundant, followed by Bacilli (16%), Actinobacteria (16%), and Bacteroidia (14%). At the order level, the microbiota was primarily composed of Micrococcales, Pseudomonadales, and Mycoplasmatales, with additional representation from Bacteroidales, Chitinophagales, and Pasteurellales (Fig. 5C). During the low-PM period, Micrococcales (19%) were dominant, followed by Pseudomonadales (12%) and Chitinophagales (9%). Mycoplasmatales and Bacteroidales were also present at 8% and 7%, respectively. During the high-PM period, Micrococcales (12%), Mycoplasmatales (11%), and Bacteroidales (8%) were most abundant. Pseudomonadales and Pasteurellales were also present at 8% and 6%, respectively. At the family level, Microbacteriaceae and Mycoplasmataceae were among the most abundant families across sampling periods (Fig. 5D). During the low-PM period, Microbacteriaceae accounted for 17% of relative abundance, followed by Moraxellaceae (12%) and Chitinophagaceae (10%), while Mycoplasmataceae accounted for 8%. During the high-PM period, Mycoplasmataceae accounted for 11%, followed by Microbacteriaceae (9%), Moraxellaceae (8%), and Pasteurellaceae (6%). At the genus level, the microbiota was primarily composed of *Moraxella* (11%), *Filobacterium* (9%), and *Mycoplasma* (8%) during the low-PM period (Fig. 5E). During the high-PM period, *Mycoplasma* was the most abundant genus (11%), followed by *Moraxella* (4%), *Filobacterium* (4%), and *Prevotella* (3%).

**Table 2:** Number of taxa detected during the low-PM and high-PM periods across different taxonomic levels

Level of Taxonomy	Low-PM period	High-PM period
Phylum	45	69
Class	116	176
Order	264	424
Family	437	705
Genus	944	1625

### Relative abundance of BRD-related and dominant commensal genera

**Fig. 6:**
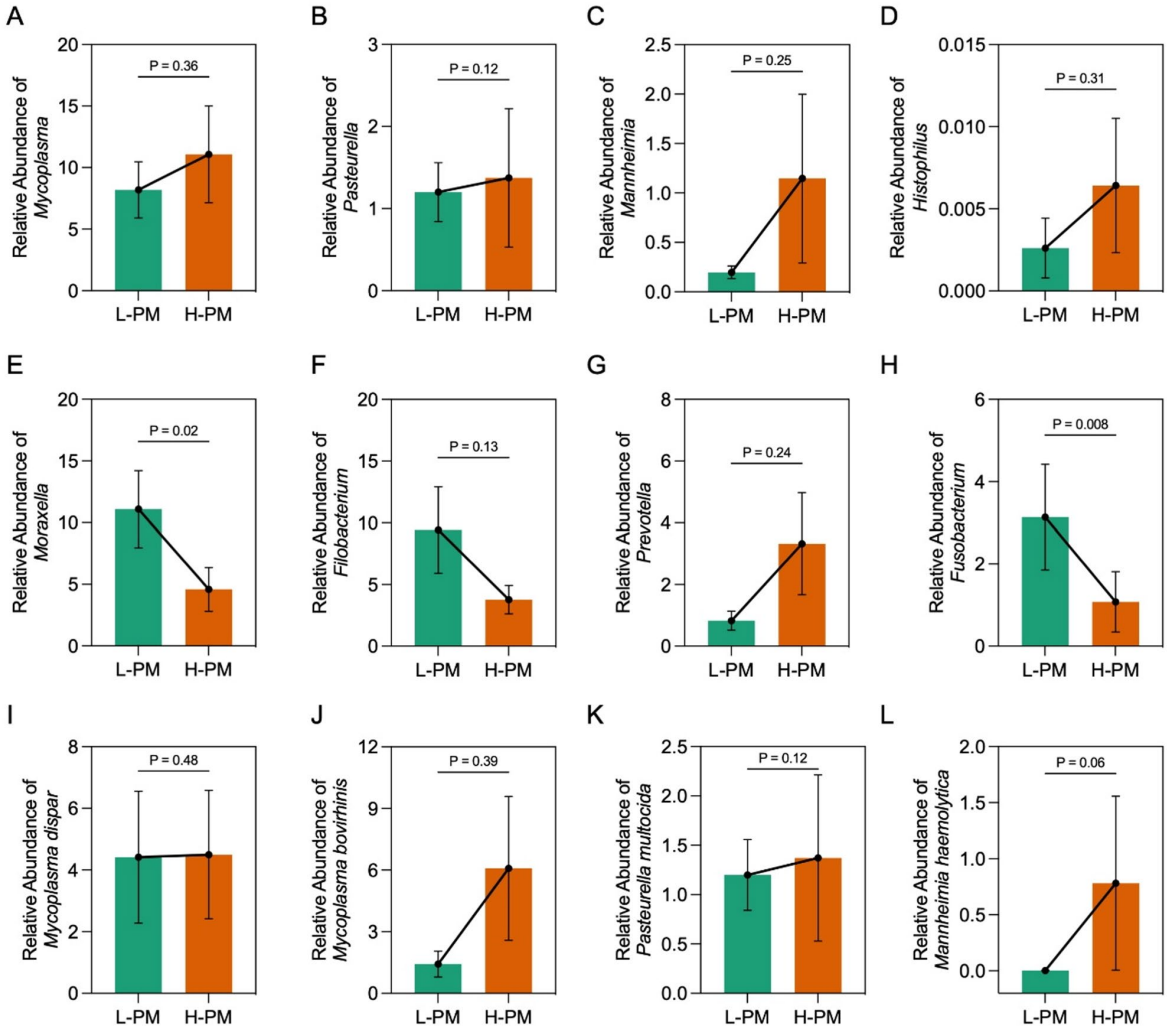
Relative abundance of selected BRD-associated and dominant commensal microbes in the nasopharyngeal microbiota of dairy heifers during the low-PM and high-PM periods. Bar plots show mean ± SEM relative abundance of (**A**) *Mycoplasma*, (**B**) *Pasteurella*, (**C**) *Mannheimia*, (**D**) *Histophilus*, (**E**) *Moraxella*, (**F**) *Filobacterium*, (**G**) *Prevotella*, (**H**) *Fusobacterium*, (**I**) *Mycoplasma dispar*, (**J**) *Mycoplasma bovirhinis*, (**K**) *Pasteurella multocida*, and (**L**) *Mannheimia haemolytica*. Connecting lines indicate changes in group mean values across sampling periods. Paired comparisons were performed using the Wilcoxon matched-pairs signed-rank test, and P values are shown in each panel. L-PM, low-PM period; H-PM, high-PM period

Further taxonomic analysis was conducted to compare the relative abundances of selected BRD-associated and dominant commensal genera between the low-PM and high-PM periods. Among the BRD-associated genera, *Mycoplasma*, *Pasteurella*, *Mannheimia*, and *Histophilus* showed higher average relative abundances during the high-PM period than during the low-PM period; however, paired comparisons were not statistically significant (Figs. 6A–D).

To further assess changes in dominant commensal genera, the relative abundances of *Moraxella*, *Filobacterium*, *Prevotella*, and *Fusobacterium* were compared between the low-PM and high-PM periods. *Moraxella* and *Fusobacterium* were significantly lower during the high-PM period than during the low-PM period (*P* < 0.05; Fig. 6E and H). *Filobacterium* also showed a lower average relative abundance during the high-PM period, whereas *Prevotella* showed a higher average relative abundance; however, these differences were not statistically significant (Figs. 6F–G).

Although species-level resolution is limited when using the V3–V4 region of the 16 S rRNA gene, several ASVs were assigned to species level with high confidence. ASVs classified as *Mycoplasma bovirhinis* (*M. bovirhinis*) and *M. haemolytica* showed higher average relative abundances during the high-PM period, *P. multocida* showed a slight increase, and *Mycoplasma dispar* (*M. dispar*) showed little change between sampling periods (Figs. 6I–L).

## Discussion

Particulate matter, particularly fine particles such as PM2.5 and PM10, is a major environmental health concern in Northern Thailand, where seasonal air pollution occurs annually during the dry season from January to April [11, 13, 14]. Chiang Mai province has experienced recurrent air pollution crises for over a decade, largely associated with biomass burning and seasonal meteorological conditions [10, 11, 16]. In the present study, dairy heifers were sampled during a low-PM period in January and again during a high-PM period in April, after approximately two months of elevated PM exposure. During the high-PM period, average PM2.5 and PM10 concentrations were markedly higher than during the low-PM period, with peak values far exceeding the WHO 24-hour air quality guideline values of 15 µg/m³ for PM2.5 and 45 µg/m³ for PM10 [1]. Because the enrolled heifers were raised in open-sided housing systems, they were likely exposed to ambient outdoor air throughout the seasonal PM episode. To our knowledge, this is the first field study to evaluate the association between naturally occurring ambient PM exposure and the upper respiratory microbiota of dairy heifers. We focused on the nasopharyngeal microbiota because it is considered an important reservoir for lower respiratory tract colonization and is closely related to respiratory health in cattle [19, 22].

Alpha diversity analysis showed that observed features, Shannon diversity, and Simpson index were all significantly higher during the high-PM period than during the low-PM period. Because Shannon diversity incorporates both richness and evenness, alpha diversity findings should be interpreted in relation to the specific metric used. Increased alpha diversity in the nasopharyngeal microbiota may reflect greater microbial richness and a potentially more stable or resilient microbial environment. However, alpha diversity should be interpreted together with taxonomic composition. In the present study, higher alpha diversity occurred alongside higher average relative abundances of BRD-associated genera, such as *Mycoplasma* and *Mannheimia*, although these differences were not statistically significant, and significant reductions in *Moraxella* and *Fusobacterium*. Similar findings from PM-exposure studies suggest that increased respiratory microbial diversity may coincide with shifts in community composition toward opportunistic or disease-associated taxa rather than representing a uniformly beneficial change [36–38]. Therefore, the higher alpha diversity observed during the high-PM period may reflect a change in respiratory microbial balance and should be interpreted cautiously.

Beta diversity analysis further indicated that the high-PM period was associated with changes in respiratory microbial community structure, although the findings were metric-dependent. Bray–Curtis dissimilarity and Jaccard distance showed significant differences between low-PM and high-PM samples, whereas unweighted UniFrac and weighted UniFrac did not. Bray–Curtis reflects differences in relative abundance, while Jaccard reflects differences in community membership based on presence–absence patterns [39, 40]. Therefore, the significant Bray–Curtis and Jaccard results suggest that the high-PM period was associated with changes in both abundance-based community structure and taxonomic membership. However, the lack of significant differences in UniFrac metrics suggests that these changes were not strongly reflected in phylogenetic community structure. Previous studies in chickens and humans have also shown that PM exposure can alter respiratory or pulmonary microbiota composition. Clear separation of microbial communities was observed between PM-exposed and control groups of chickens, indicating a substantial compositional shift in pulmonary microbiota due to PM exposure [41]. Similarly, human studies have reported changes in airway or pharyngeal microbiota composition associated with ambient PM exposure [37, 38]. Thus, our beta-diversity findings support the interpretation that the high-PM period was associated with respiratory microbiota restructuring, particularly in terms of taxonomic composition and relative abundance.

In the present study, the respiratory microbiota was dominated by Proteobacteria, Actinobacteriota, Firmicutes, and Bacteroidota. Relative abundance profiles showed changes in the proportional composition of higher-level taxa between the low-PM and high-PM periods. During the high-PM period, Proteobacteria and Firmicutes accounted for higher proportions of the microbiota, whereas Actinobacteriota and Bacteroidota accounted for lower proportions. At the class level, Gammaproteobacteria and Bacilli accounted for higher proportions during the high-PM period, while Actinobacteria and Bacteroidia accounted for lower proportions. These phylum- and class-level patterns provide an overview of community composition across sampling periods. Previous studies in cattle have reported Proteobacteria and Firmicutes as major phyla in the upper respiratory tract of beef and feedlot cattle, including cattle with respiratory disease [42, 43]. Similar compositional patterns have also been reported in chickens and humans exposed to air pollution, including higher proportions of Proteobacteria and lower proportions of Bacteroidota or Bacteroidia [7, 41]. Together, these observations suggest that the high-PM period was accompanied by changes in the overall taxonomic profile of the respiratory microbiota.

At the order and family levels, the high-PM period was characterized by higher proportions of Mycoplasmatales, Bacteroidales, Pasteurellales, Mycoplasmataceae, and Pasteurellaceae, and lower proportions of Micrococcales, Chitinophagales, and Microbacteriaceae. These patterns are relevant to respiratory microbiota ecology because Mycoplasmataceae and Pasteurellaceae include several genera commonly detected in the bovine upper respiratory tract and implicated in BRD, including *Mycoplasma*, *Mannheimia*, *Pasteurella*, and *Histophilus* [20–22]. Bacteroidales, in contrast, should be interpreted more broadly as part of the respiratory microbial community, including anaerobic taxa such as Prevotella, rather than as a direct BRD-associated group. These order- and family-level profiles further support the interpretation that the high-PM period was accompanied by changes in the overall taxonomic composition of the respiratory microbiota.

At the genus level, *Moraxella*, *Filobacterium*, and *Mycoplasma* were among the dominant genera during the low-PM period, whereas *Mycoplasma* accounted for the highest proportion during the high-PM period. Among the selected dominant genera, *Moraxella* and *Fusobacterium* were significantly lower during the high-PM period than during the low-PM period. The decline in *Moraxella* is particularly relevant because this genus is commonly reported as a predominant member of the healthy bovine upper respiratory microbiota. A high abundance of *Moraxella* has been observed at both nostril and nasopharyngeal sites in healthy feedlot cattle, suggesting that it may represent a core member of the normal bovine respiratory microbiota [19]. Moreover, *Moraxella bovoculi* was reported to be more abundant in healthy calves than in calves with bovine pneumonia, supporting its possible association with respiratory microbial stability [44]. Human data have also shown that air pollution exposure may be associated with reduced *Moraxella* abundance in the respiratory tract [7]. Therefore, the significant reduction in *Moraxella* during the high-PM period may indicate a loss of a dominant commensal component of the bovine respiratory microbiota.

*Fusobacterium* was also significantly lower during the high-PM period. Although *Fusobacterium* includes species that may act as opportunists in some anatomical sites [45], its role in the bovine upper respiratory microbiota is less clearly defined than that of *Moraxella*. In the present study, the reduction in *Fusobacterium* should therefore be interpreted as part of the broader restructuring of the respiratory microbial community rather than as direct evidence of improved or impaired respiratory health. In contrast, *Filobacterium* showed a lower average relative abundance and *Prevotella* showed a higher average relative abundance during the high-PM period, but these differences were not statistically significant.

Selected BRD-associated genera, including *Mycoplasma*, *Pasteurella*, *Mannheimia*, and *Histophilus*, were evaluated because of their relevance to bovine respiratory disease and their potential presence in the upper respiratory tract of clinically healthy cattle [20–22]. In the present study, these genera showed higher average relative abundances during the high-PM period than during the low-PM period; however, paired comparisons were not statistically significant. Therefore, these findings do not provide conclusive evidence that the high-PM period increased BRD-associated genera. Nevertheless, the higher average relative abundances observed during the high-PM period may warrant further investigation in larger longitudinal studies, particularly because BRD is a multifactorial disease involving environmental stressors, host immunity, and microbial imbalance [20, 21].

At the species level, ASVs classified as *M. bovirhinis* represented the most abundant species-level assignment during the high-PM period and showed a higher average relative abundance than during the low-PM period. ASVs classified as *M. haemolytica* also showed higher average relative abundance during the high-PM period, *P. multocida* showed a slight increase, and *M. dispar* showed little change between sampling periods. These observations are relevant because these species have been associated with the bovine respiratory tract and BRD. In addition, *Mycoplasma* was the most abundant genus during the high-PM period, and previous studies have reported *Mycoplasma* species, particularly *M. dispar* and *M. bovirhinis*, as common members of the bovine nasopharyngeal microbiota during management stress and BRD-related contexts [46, 47]. Together, these findings suggest that the high-PM period may be associated with a respiratory microbiota profile containing higher average proportions of BRD-relevant taxa, although further studies are needed to determine whether these changes contribute to BRD susceptibility.

Air pollution may act as a predisposing environmental factor for respiratory microbiota dysbiosis, although its direct contribution to BRD development requires further investigation. Together with the significant reductions in *Moraxella* and *Fusobacterium*, increased alpha diversity, and significant Bray–Curtis and Jaccard differences, these findings suggest that the high-PM period was associated with respiratory microbiota restructuring in clinically healthy heifers.

Supporting evidence suggests that PM2.5 can alter respiratory microbiota composition, increase bacterial load, and enhance host susceptibility to respiratory infections [24, 26]. PM exposure may disrupt host–microbe interactions [48]. In poultry models, PM2.5-induced dysbiosis has been linked to lung inflammation and injury, and microbiota-targeted intervention reduced lung inflammation, supporting a functional link between PM-associated microbial imbalance and respiratory pathology [9]. However, because the present study did not directly assess host immune responses, lung pathology, or clinical BRD outcomes, these mechanisms should be interpreted as biologically plausible explanations rather than direct evidence from the enrolled heifers.

Several limitations should be considered when interpreting these findings. First, this was a natural field study without an unexposed indoor control group; therefore, the observed microbiota changes cannot be attributed solely to PM exposure. Other seasonal or farm-level factors, including temperature, humidity, feeding, bedding, barn airflow, and animal management events, may also have contributed. Second, the study included a limited number of replacement dairy heifers, and the findings may not be generalizable to older, lactating, or multiparous cows. Finally, host immune responses, lung pathology, and clinical BRD outcomes were not assessed; therefore, the health implications of the observed microbiota changes require further investigation.

Overall, the present study suggests that seasonal high-PM exposure in Northern Thailand was associated with measurable changes in the bovine nasopharyngeal microbiota. The high-PM period was characterized by increased alpha diversity, significant differences in Bray–Curtis and Jaccard beta diversity, altered taxonomic profiles, and significant reductions in *Moraxella* and *Fusobacterium*. Although BRD-associated genera showed higher average relative abundances during the high-PM period, these differences were not statistically significant. These findings support the hypothesis that ambient air pollution may contribute to respiratory microbiota restructuring in dairy heifers and highlight the need for future studies integrating microbiota composition, host immune responses, farm-level environmental monitoring, and clinical respiratory outcomes.

## Conclusions

This study suggests that seasonal high-PM exposure in Northern Thailand was associated with measurable changes in the nasopharyngeal microbiota of dairy heifers. Compared with the low-PM period, the high-PM period was characterized by significantly increased alpha diversity, significant differences in Bray–Curtis and Jaccard beta diversity, altered taxonomic profiles, and significant reductions in *Moraxella* and *Fusobacterium*. BRD-associated genera, including *Mycoplasma*, *Pasteurella*, *Mannheimia*, and *Histophilus*, showed higher average relative abundances during the high-PM period; however, these differences were not statistically significant. These findings indicate that ambient air pollution may contribute to respiratory microbiota restructuring in clinically healthy dairy heifers. Further longitudinal studies integrating microbiota composition, host immune responses, farm-level environmental monitoring, and clinical respiratory outcomes are needed to clarify whether PM-associated microbiota changes contribute to BRD susceptibility.

## Supplementary Information

Supplementary Material 1.

## Data Availability

The datasets generated during the current study are available in the NCBI repository under BioProject accession number PRJNA1298536 (http://www.ncbi.nlm.nih.gov/bioproject/1298536).
