# Supplementary material for: Seasonal particulate matter exposure is associated with upper respiratory microbiota restructuring in dairy heifers
Source: BMC Vet Res. 2026 May 22;22:424. doi: 10.1186/s12917-026-05580-y (PMC13371544; doi:10.1186/s12917-026-05580-y)
Supplement: Supplementary file 1 — Supplementary Material 1. [file 12917_2026_5580_MOESM1_ESM.docx]

**Supplementary Data**

**Supplementary Table S1.** Sample-level DADA2 denoising statistics for 16S rRNA gene amplicon sequencing of nasopharyngeal samples.

| sample-id | input | filtered | percentage of input passed filter | denoised | merged | percentage of input merged | non-chimeric | percentage of input non-chimeric |
| --- | --- | --- | --- | --- | --- | --- | --- | --- |
| A01 | 71260 | 67032 | 94.07 | 65791 | 60565 | 84.99 | 59946 | 84.12 |
| A02 | 62289 | 58475 | 93.88 | 57397 | 54034 | 86.75 | 52576 | 84.41 |
| A03 | 101249 | 89957 | 88.85 | 88316 | 81229 | 80.23 | 80650 | 79.66 |
| A04 | 192934 | 182108 | 94.39 | 180507 | 177770 | 92.14 | 174458 | 90.42 |
| A05 | 110327 | 103755 | 94.04 | 102077 | 94243 | 85.42 | 92699 | 84.02 |
| A06 | 75710 | 69384 | 91.64 | 68765 | 67196 | 88.75 | 67150 | 88.69 |
| A07 | 187715 | 177477 | 94.55 | 177074 | 176159 | 93.84 | 175946 | 93.73 |
| A08 | 246069 | 231241 | 93.97 | 229128 | 216424 | 87.95 | 214263 | 87.07 |
| A09 | 211689 | 199740 | 94.36 | 198876 | 196906 | 93.02 | 195772 | 92.48 |
| A10 | 112152 | 105763 | 94.3 | 104572 | 101219 | 90.25 | 99847 | 89.03 |
| A12 | 131870 | 123345 | 93.54 | 122248 | 118460 | 89.83 | 117537 | 89.13 |
| A14 | 94874 | 89852 | 94.71 | 89562 | 89087 | 93.9 | 88852 | 93.65 |
| A18 | 121747 | 114505 | 94.05 | 111502 | 102508 | 84.2 | 95923 | 78.79 |
| A19 | 132494 | 125189 | 94.49 | 124757 | 124136 | 93.69 | 123940 | 93.54 |
| A20 | 248921 | 235132 | 94.46 | 231987 | 222646 | 89.44 | 216086 | 86.81 |
| A21 | 150068 | 140878 | 93.88 | 139060 | 130393 | 86.89 | 128028 | 85.31 |
| A22 | 206407 | 195289 | 94.61 | 191753 | 180020 | 87.22 | 159245 | 77.15 |
| A23 | 147585 | 139277 | 94.37 | 134910 | 120299 | 81.51 | 108029 | 73.2 |
| A24 | 97262 | 91068 | 93.63 | 89640 | 81758 | 84.06 | 80042 | 82.3 |
| A25 | 168482 | 158843 | 94.28 | 157553 | 151798 | 90.1 | 150395 | 89.26 |
| A26 | 168483 | 157628 | 93.56 | 156056 | 150225 | 89.16 | 148165 | 87.94 |
| A27 | 99842 | 93365 | 93.51 | 93004 | 92157 | 92.3 | 92090 | 92.24 |
| A28 | 187521 | 162934 | 86.89 | 160021 | 116972 | 62.38 | 113729 | 60.65 |
| A29 | 205789 | 192853 | 93.71 | 190506 | 180024 | 87.48 | 178880 | 86.92 |
| A30 | 87343 | 81783 | 93.63 | 80271 | 71395 | 81.74 | 68615 | 78.56 |
| B01 | 144935 | 135628 | 93.58 | 133882 | 122687 | 84.65 | 120725 | 83.3 |
| B02 | 222984 | 209219 | 93.83 | 206365 | 186026 | 83.43 | 180917 | 81.13 |
| B03 | 122430 | 107787 | 88.04 | 104930 | 86470 | 70.63 | 85253 | 69.63 |
| B04 | 54453 | 50481 | 92.71 | 48999 | 41082 | 75.44 | 40500 | 74.38 |
| B05 | 74477 | 70137 | 94.17 | 68728 | 60931 | 81.81 | 59960 | 80.51 |
| B06 | 207123 | 182242 | 87.99 | 179653 | 139448 | 67.33 | 138900 | 67.06 |
| B07 | 116149 | 108857 | 93.72 | 108177 | 107273 | 92.36 | 99761 | 85.89 |
| B08 | 323731 | 288098 | 88.99 | 270584 | 215264 | 66.49 | 193436 | 59.75 |
| B09 | 108997 | 102970 | 94.47 | 102115 | 100454 | 92.16 | 96752 | 88.77 |
| B10 | 316937 | 296150 | 93.44 | 292784 | 265738 | 83.85 | 263591 | 83.17 |
| B12 | 188944 | 165300 | 87.49 | 161864 | 129403 | 68.49 | 127065 | 67.25 |
| B14 | 77685 | 73414 | 94.5 | 72944 | 72168 | 92.9 | 71871 | 92.52 |
| B18 | 128956 | 114862 | 89.07 | 111641 | 93255 | 72.32 | 88486 | 68.62 |
| B19 | 169020 | 159016 | 94.08 | 157272 | 147977 | 87.55 | 145583 | 86.13 |
| B20 | 302693 | 265595 | 87.74 | 249848 | 204567 | 67.58 | 182629 | 60.33 |
| B21 | 60804 | 57889 | 95.21 | 57538 | 56831 | 93.47 | 56583 | 93.06 |
| B22 | 109596 | 103654 | 94.58 | 101044 | 93306 | 85.14 | 86280 | 78.73 |
| B23 | 100922 | 95045 | 94.18 | 94390 | 93018 | 92.17 | 92387 | 91.54 |
| B24 | 72920 | 69604 | 95.45 | 68010 | 64990 | 89.13 | 63076 | 86.5 |
| B25 | 860442 | 808928 | 94.01 | 804022 | 761610 | 88.51 | 746930 | 86.81 |
| B26 | 199525 | 187584 | 94.02 | 186106 | 182773 | 91.6 | 163463 | 81.93 |
| B27 | 115341 | 108116 | 93.74 | 106634 | 97440 | 84.48 | 95661 | 82.94 |
| B28 | 304469 | 287288 | 94.36 | 284129 | 264283 | 86.8 | 134771 | 44.26 |
| B29 | 232856 | 219081 | 94.08 | 215882 | 195265 | 83.86 | 102552 | 44.04 |
| B30 | 489401 | 460820 | 94.16 | 458084 | 422352 | 86.3 | 227332 | 46.45 |


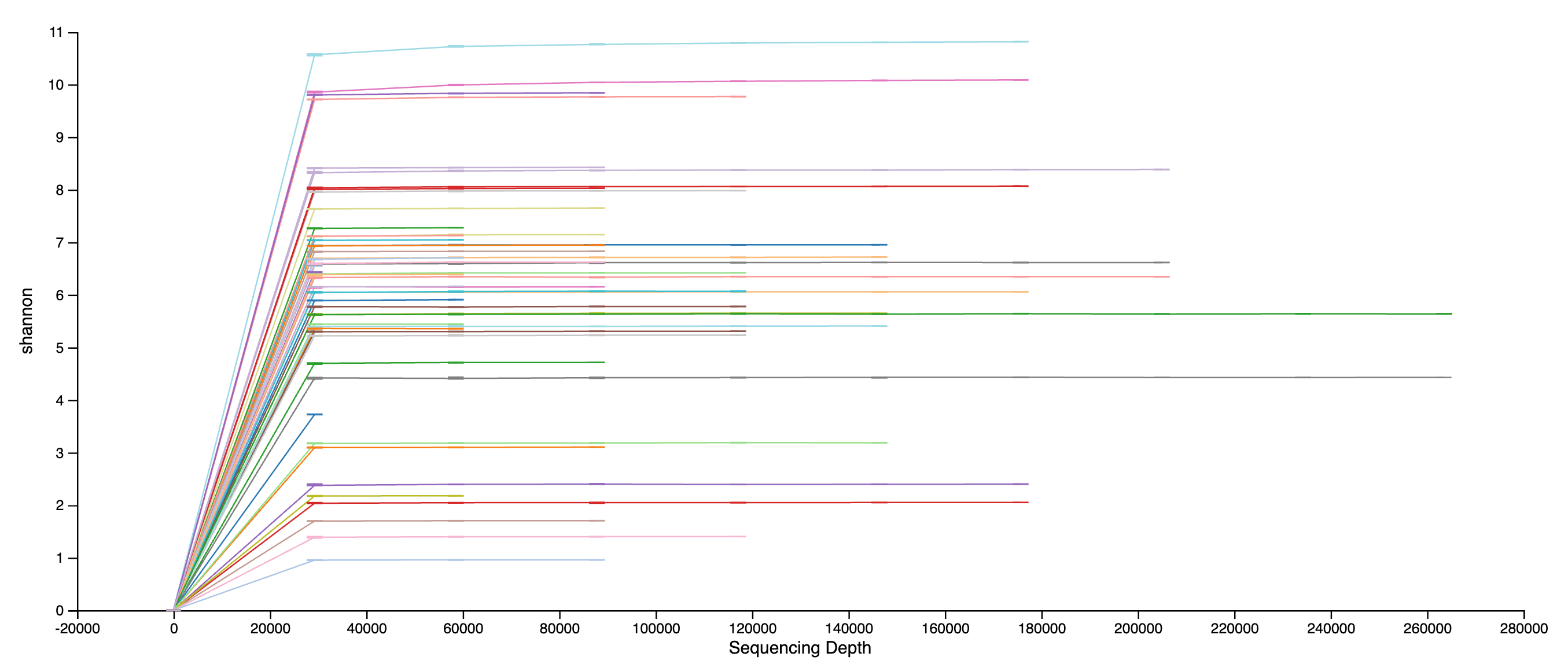


**Supplementary Figure S1**. Alpha rarefaction curves of nasopharyngeal microbiota samples. Rarefaction analysis was performed to assess sequencing depth across samples.
